# Supplementary material for: Design of a self-regulating mRNA gene circuit
Source: Sci Rep. 2024 Aug 21;14:19421. doi: 10.1038/s41598-024-70363-0 (PMC11339067; doi:10.1038/s41598-024-70363-0)
Supplement: Supplementary file 1 — Supplementary Information. [file 41598_2024_70363_MOESM1_ESM.pdf]

Supplementary Data

Eric C. Dykeman

Plasmids and primer sequences

DNA sequence constructs containing the CTnano and CTnano-cnt mRNAs used in molecular cloning are given below. The DNA sequences were inserted into the pET-21a(+) vector (Novagen) using restriction sites BgIII and Bpu1102. Supplementary Figure 1 gives a general diagram of the plasmids pET-CTnano and pET-CTnano-cnt.

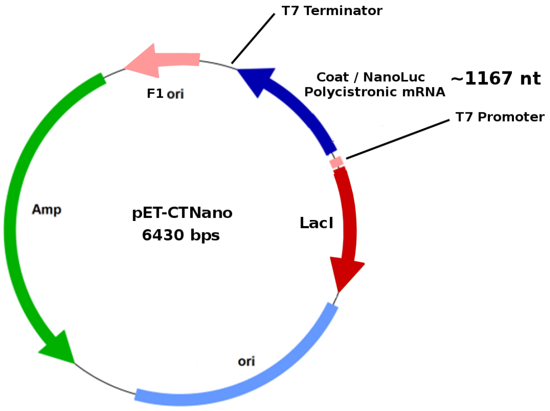

Sup. Figure 1. Plasmid diagram of pET-CTnano.

Primers for Q5 mutagenesis. Primers and PCR settings used for mutagenesis of the pET-CTnano and pET-CTnano-cnt plasmids are listed in Supplementary Table 1. Amplification of the original plasmid DNA was performed using the Q5 polymerase supplied from the NEB Q5-mutagenesis kit (New England Biolabs) and the primers listed in Sup. Table 1. The PCR reaction was started with denaturation for 1 min at 98° C, followed by twenty-five rounds of PCR amplification. One amplification round consisted of (1) denaturation for 10 sec at 98° C, (2) primer hybridization for 30 sec at T_a, (3) polymerase chain extension for 2 min 20 sec at 72° C. The PCR reaction was then finished with a final extension at 72° C for 2 min.

Sup. Table 1. Primers and PCR settings used in mutagenesis. Primer melting and annealing temperatures are given by the NEB base changer tool (https://nebasechanger.neb.com) in degrees C.

Primer Name	Coat Mutation	Primer Sequence	T_m	T_a
for-ms2nl-mut2	N55D	CTCTGCGCAGGATCGCAAATA	64	63
rev-ms2nl-mut2	N55D	CTCTGACGAACGCTACAG	62	63
for-ms2nl-mut3	T19*	AACTGGCGACTAGTAAGTCGCCCCAAGCAACTTC	63	64
rev-ms2nl-mut3	T19*	CCGCCATTGTCGACGAGA	67	64
for-ms2nl-mut7	S37*	CAGCTCTAACTAGCGTTCACAGG	65	66
rev-ms2nl-mut7	S37*	ATCCATTCAGCGACCCCG	68	66

Predicted secondary structures of CTnano-cnt and CTnano mRNAs

Predicted secondary structures of the CTnano-cnt and CTnano mRNAs were computed using standard prediction tools for the thermodynamic minimum free energy. The long-distance Min Jou interaction was enforced, assuming that co-transcriptional folding would result in the folding of the coat domain and Min Jou interaction by the time the nano luciferase domain was reached by the polymerase. Supplementary Figure 2 shows the structure of CTnano-cnt mRNA, while Sup. Figure 3 shows the structure of the CTnano mRNA along with simplified cartoon diagrams.

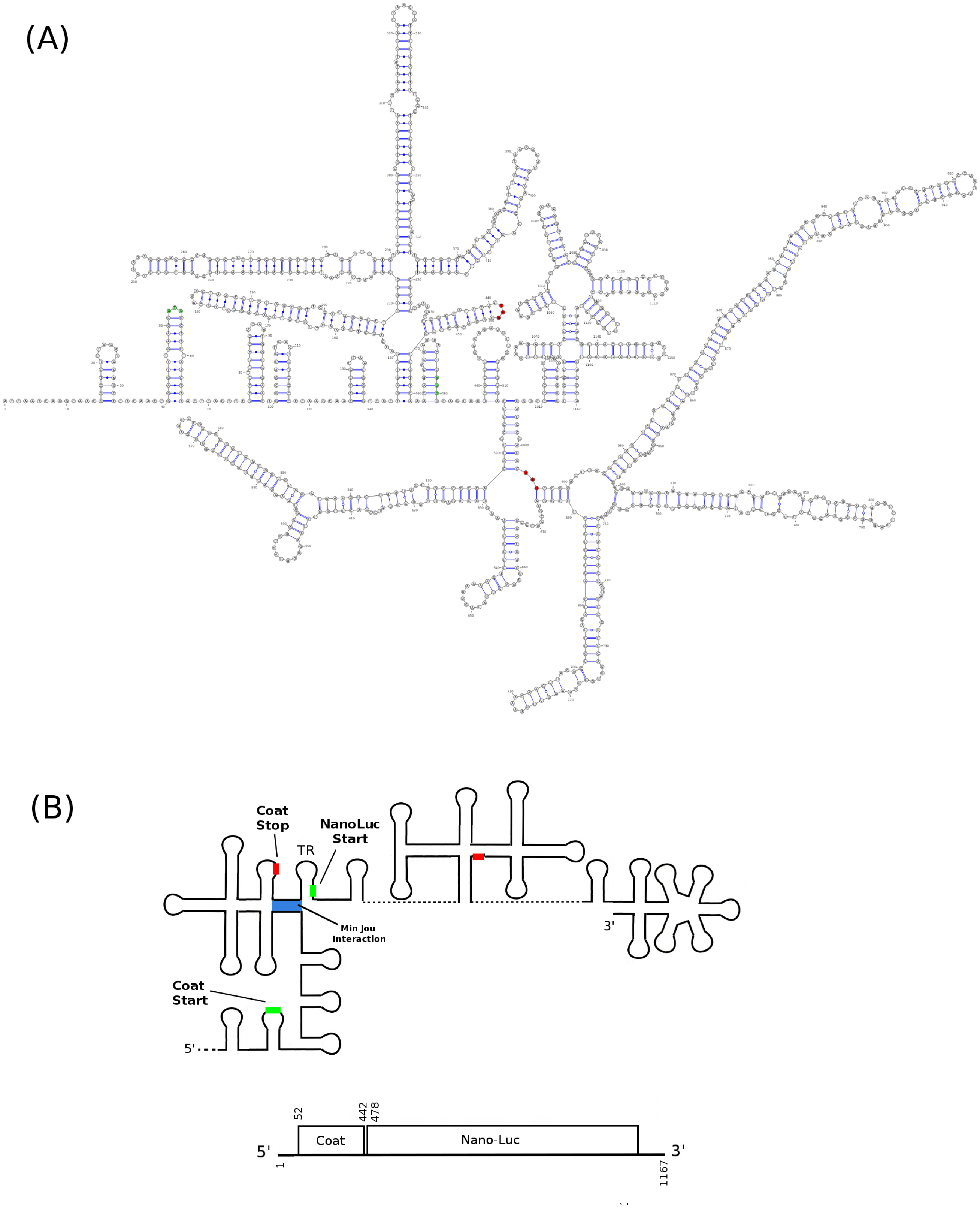

Sup. Figure 2. Predicted secondary structure of CTnano-cnt mRNA.

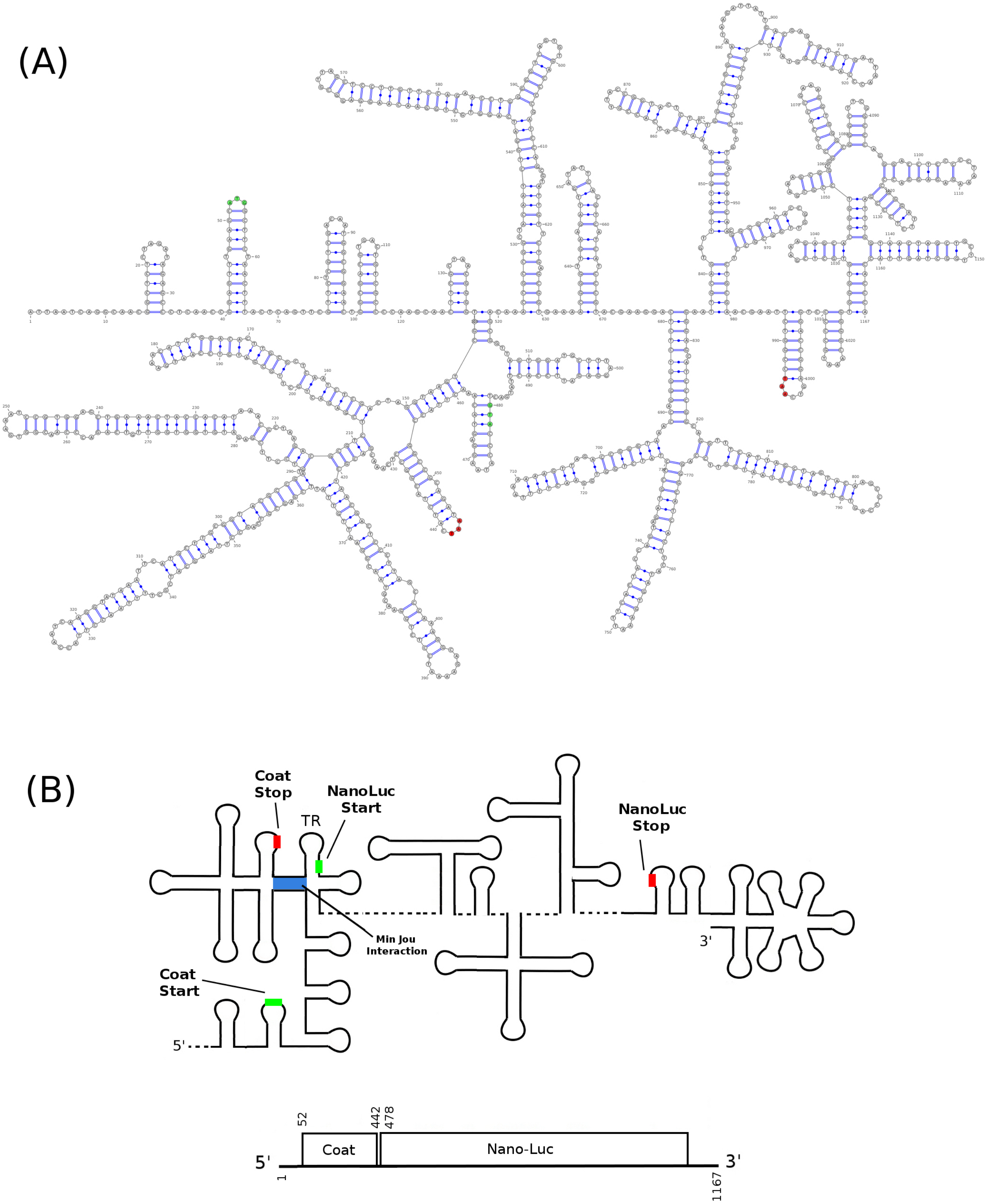

Sup. Figure 3. Predicted secondary structure of CTnano mRNA.

Stop codon mutations to CTnano-cnt and CTnano mRNAs

Supplementary Figure 4 shows the relative positions of T19* and S37* mutations relative to the position of the Min Jou Sequence.

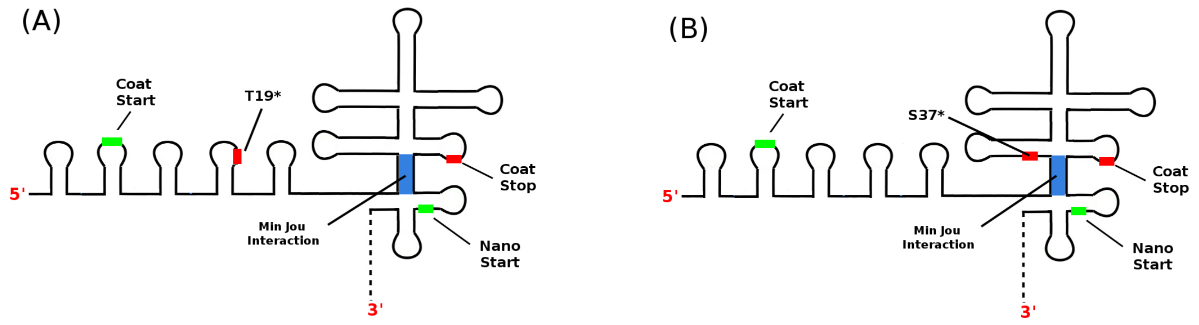

Sup. Figure 4. The relative positions of T19* and S37* mutations to the position of the Min Jou Sequence. (A) Position of the T19* codon mutation, which is directly upstream of the Min Jou long-distance interaction (highligted blue). Ribsomes reaching this stop codon should fail to disrupt the Min Jou interaction. (B) Position of the S37* codon mutation which is directly downstream of the Min Jou long-distance interaction. Ribosomes reaching this stop codon will have fully disrupted the Min Jou interaction.

Experimental time course of NanoGlo assay

Experimental time courses for the NanoGlo assay are shown for CTnano-cnt mRNA (Sup. Figure 5) and CTnano (Sup. Figure 6) below. Measurements were performed on three culture samples and error bars are the calculated standard deviation of the measurements. Data points were normalised by taking the measured luminescence and dividing by the cultures A_{600} value.

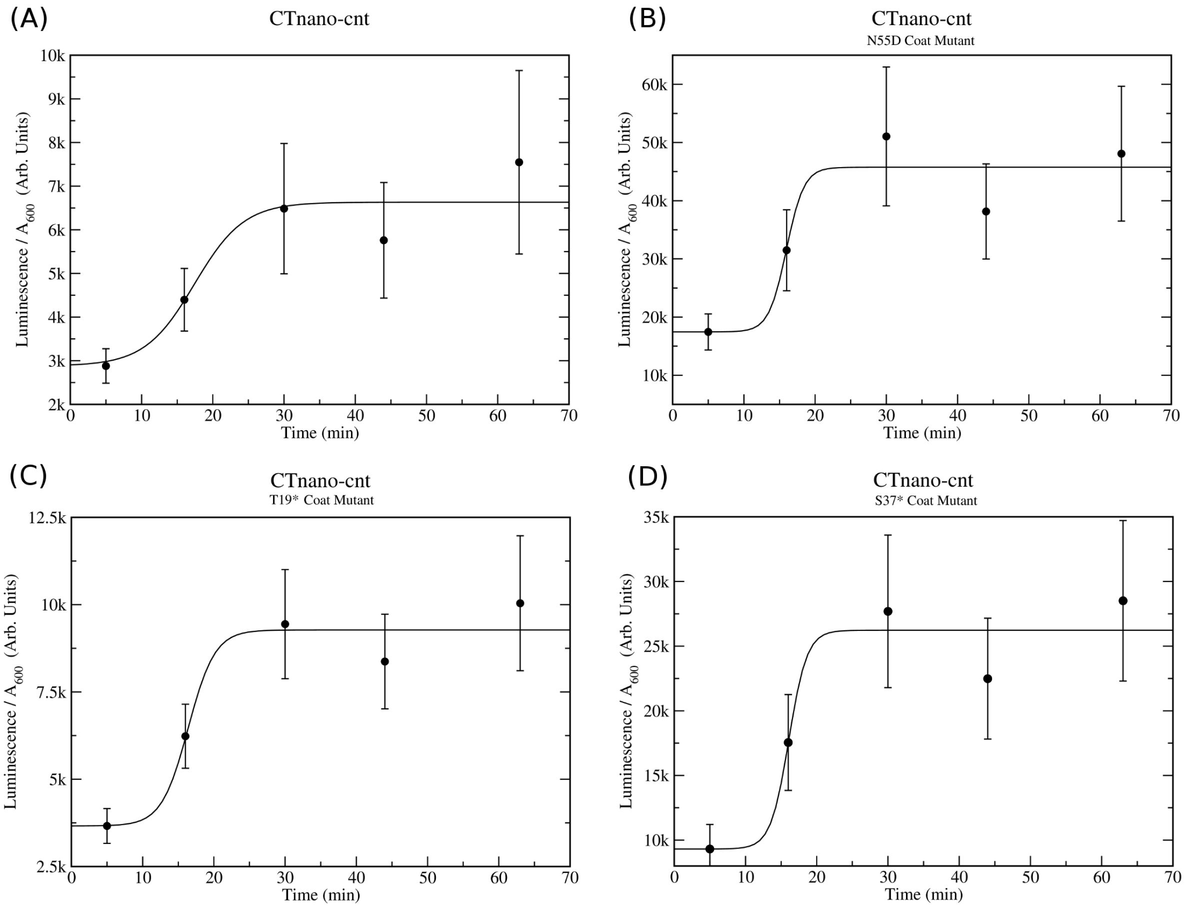

Sup. Figure 5. Experimental time course of NanoGlo Assay on CTnano-cnt mRNA and its mutants. Measurements were performed following the procedure in methods and data points are fitted to the equation $L(t) = a + b/(1 + exp(-c(t - d)))$, where a, b, c and d are constants. NanoGlo assays for CTnano-cnt with (A) wild-type MS2 coat protein, (B) N55D coat mutant, (C) T19* coat mutant, and (D) S37* coat mutant.

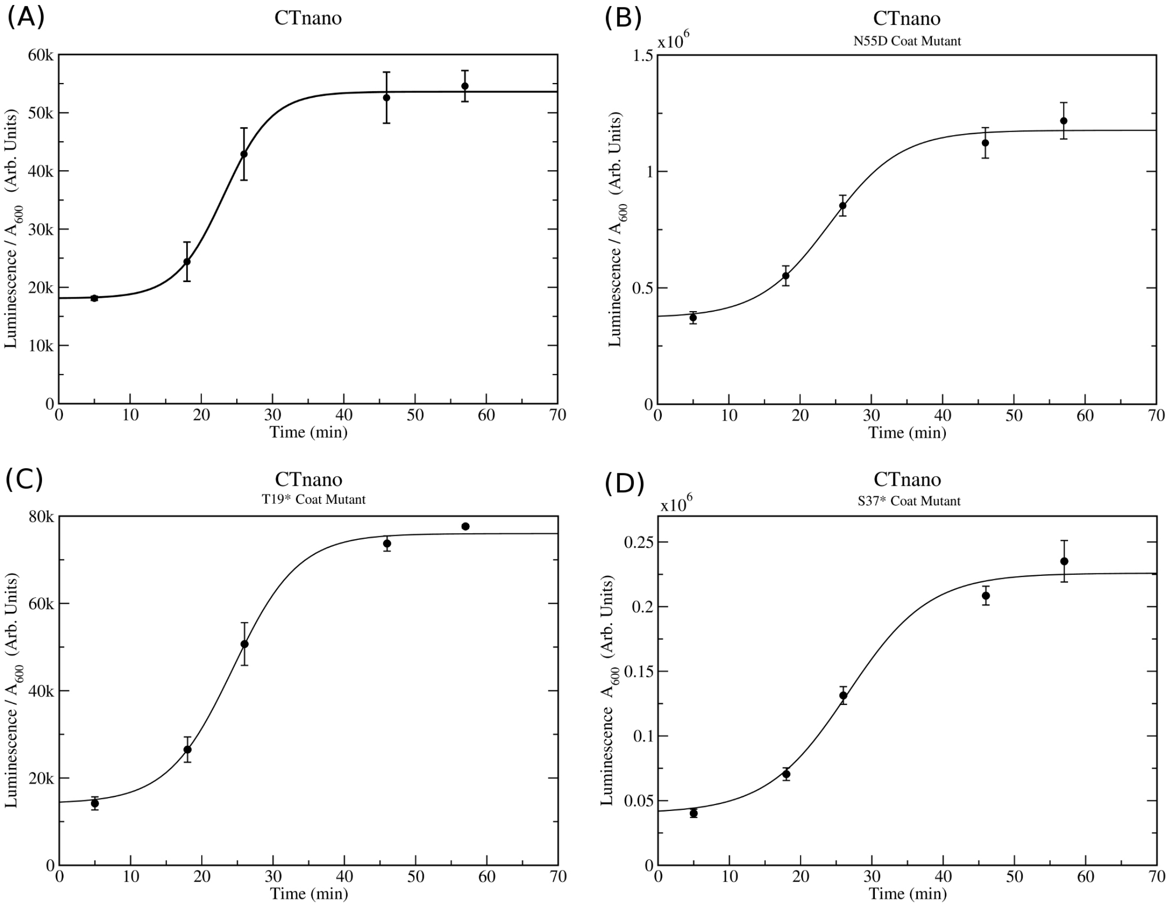

Sup. Figure 6. Experimental time course of NanoGlo Assay on CTnano mRNA and its mutants. Measurements were performed following the procedure in methods and data points are fitted to the equation $L(t) = a + b/(1 + exp(-c(t - d)))$, where a, b, c and d are constants. NanoGlo assays for CTnano with (A) wild-type MS2 coat protein, (B) N55D coat mutant, (C) T19* coat mutant, and (D) S37* coat mutant.

Co-translational folding kinetics of the CTnano-cnt NLuc ribosome binding site

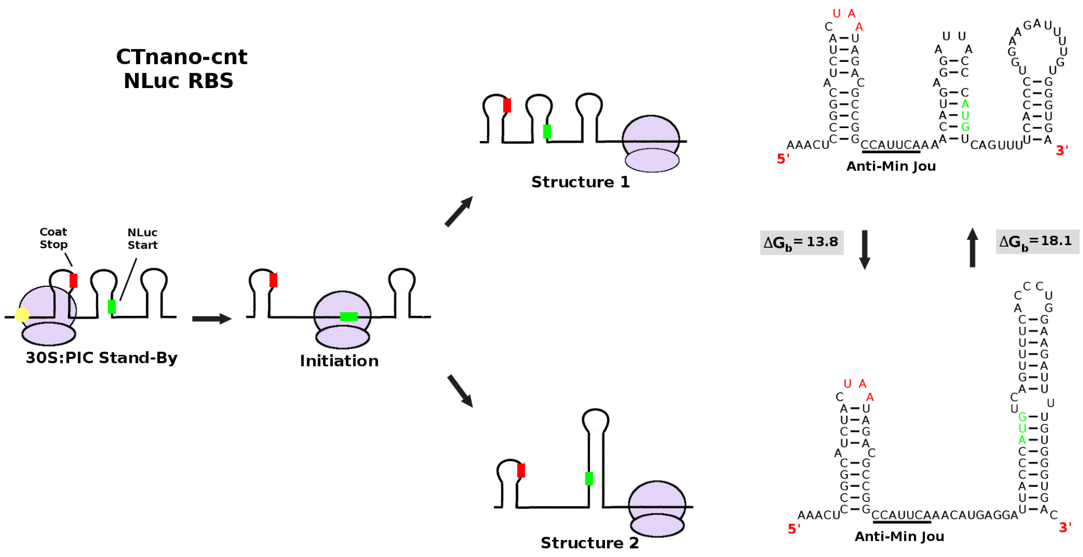

Sup. Figure 7. Cartoon diagram illustrating the co-translational folding kinetics of the NLuc ribosome binding site (RBS) in the CTnano-cnt mRNA. Two alternative folding pathways for the RBS can occur as the 5' RNA re-folds during ribosome translation. This results in either the folding of the TR stem-loop (Structure 1), or the folding of an alternative more stable hairpin (Structure 2). The energetic barrier to transition from structure 1 to structure 2 is estimated at $\Delta G_b = 13.8$ kcal/mol. When compared to the barrier between structure 2 and structure 1 ($\Delta G_b = 18.1$ kcal/mol), it is likely structure 2 represents a long lived kinetic trap.

Computational files and commands for protein simulations

The computation simulations for protein synthesis where performed for the optimised mRNA by executing the following command with the files ms2__K12__T10.dat and ms2__nluc__opt.dat (provided in supplementary material)

```
./Prosyn.x -x 25.0 -s mrna_K12_T10.dat -v ms2_nluc_opt.fasta -o output -t 1800.0
```

Similarly, the simulations for protein synthesis where performed for the optimised mRNA by executing the following command with the files ms2__K12__T10.dat and ms2__nluc__cnt.dat

```
./Prosyn.x -x 25.0 -s mrna_K12_T10.dat -v ms2_nluc_cnt.fasta -o output -t 1800.0
```

After running these commands, data for the coat protein and nanoluc numbers are then output in the final two columns in the ribo__dependent__pro.dat file.

Sequences ordered for plasmid preparation

>CTnano-cnt
AAAGGGAAAGGGAGATCTAATACGACTCACTATAGGGATTAATCAGGCAACGGCTCTCTA
GATAGAGCCCTCAACCGGAGTTTGAAGCATGGCTTCTAACTTTACTCAGTTCGTTCTCGT
CGACAATGGCGGAACTGGCGACGTGACTGTCGCCCCAAGCAACTTCGCTAACGGGGTCGC
TGAATGGATCAGCTCTAACTCGCGTTCACAGGCTTACAAAGTAACCTGTAGCGTTCGTCA
GAGCTCTGCGCAGAATCGCAAATACACCATCAAAGTCGAGGTGCCTAAAGTGGCAACCCA
GACTGTTGGTGGTGTAGAGCTTCCTGTAGCCGCATGGCGTTCGTACTTAAATATGGAACT
AACCATTCCAATTTTCGCTACGAATTCCGACTGCGAGCTTATTGTTAAGGCAATGCAAGG
TCTCCTAAAAGACGGAAACCCGATTCCCTCAGCAATCGCAGCAAACTCCGGCATCTACTA
ATAGACGCCGGCCATTCAAACATGAGGATTACCCATGTCAGTTTTCACCCTGGAAGATTT
TGTGGGTGACTGGCGTCAGACCGCCGGCTATAATCTGGATCAGGTGCTGGAACAGGGTGG
CGTTAGTAGTCTGTTTCAGAATCTGGGTGTGAGTGTGACCCCGATTCAGCGTATTGTTCT
GAGCGGTGAAAATGGTCTGAAAATTGATATTCATGTGATCATTCCGTACGAAGGCCTGAG
TGGTGACCAGATGGGTCAGATTGAAAAAATTTTTAAGGTGGTTTACCCGGTGGATGATCA
TCATTTTAAAGTGATTCTGCATTACGGAACGCTGGTTATTGATGGTGTGACCCCGAATAT
GATTGATTATTTTGGCCGTCCGTATGAAGGTATTGCAGTGTTTGATGGCAAAAAGATTAC
CGTTACCGGAACGCTGTGGAATGGCAATAAGATTATTGATGAACGTCTGATTAACCCGGA
TGGTAGTCTGCTGTTTCGTGTGACCATTAATGGCGTTACCGGTTGGCGTCTGTGCGAACG

CATTCTGGCCTAACTGAGGCCAGGTCCCCCGTAAACGGGGTGGGTGTGCTCGAAAGAGCA
CGGGTCCGCGAAAGCGGTGGCTCCACCGAAAGGTGGGCGGGCTTCGGCCCAGGGACCTCC
CCCTAAAGAGAGGACCCGGGATTCTCCCGATTTGGTAACTAGCTGCTTGGCTAGTTACCA
CCCACTAGCATAACCCCTTGGGGCCTCTAAACGGGTCTTGAGGGGTTTTTTGGTACCAAA
GGCTGAGCAAAGGG
>CTnano
AAAGGGAAAGGGAGATCTAATACGACTCACTATAGGGATTAATCAGGCAACGGCTCTCTA
GATAGAGCCCTCAACCGGAGTTTGAAGCATGGCTTCTAACTTTACTCAGTTCGTTCTCGT
CGACAATGGCGGAACTGGCGACGTGACTGTCGCCCCAAGCAACTTCGCTAACGGGGTCGC
TGAATGGATCAGCTCTAACTCGCGTTCACAGGCTTACAAAGTAACCTGTAGCGTTCGTCA
GAGCTCTGCGCAGAATCGCAAATACACCATCAAAGTCGAGGTGCCTAAAGTGGCAACCCA
GACTGTTGGTGGTGTAGAGCTTCCTGTAGCCGCATGGCGTTCGTACTTAAATATGGAACT
AACCATTCCAATTTTCGCTACGAATTCCGACTGCGAGCTTATTGTTAAGGCAATGCAAGG
TCTCCTAAAAGACGGAAACCCGATTCCCTCAGCAATCGCAGCAAACTCCGGCATCTACTA
ATAGACGCCGGCCATTCAAACATGAGGAATACCCATGTCAGTATTCACCTTAGAGGATTT
CGTAGGTGATTGGCGACAAACCGCCGGCTACAATCTGGATCAGGTGCTGGAACAAGGAGG
CGTTAGCTCCTTGTTCCAGAACCTGGGGGTCAGTGTGACCCCGATCCAGCGTATTGTTCT
GAGCGGTGAAAACGGGTTGAAGATTGATATTCACGTCATCATCCCGTACGAAGGACTCTC
TGGGGACCAAATGGGCCAGATTGAAAAAATTTTCAAGGTGGTCTATCCCGTAGATGACCA
TCACTTTAAAGTGATACTTCACTACGGGACCTTGGTAATCGATGGTGTGACCCCGAACAT
GATCGATTACTTTGGACGGCCCTACGAGGGAATTGCAGTGTTTGATGGCAAAAAGATCAC
CGTTACCGGTACTCTTTGGAACGGCAATAAGATTATTGACGAGCGTCTGATTAACCCAGA
CGGTAGTCTGCTGTTCCGTGTGACCATCAACGGCGTCACCGGTTGGCGCCTCTGCGAACG
AATTCTGGCCTAACTGAGGCCAGGTCCCCCGTAAACGGGGTGGGTGTGCTCGAAAGAGCA
CGGGTCCGCGAAAGCGGTGGCTCCACCGAAAGGTGGGCGGGCTTCGGCCCAGGGACCTCC
CCCTAAAGAGAGGACCCGGGATTCTCCCGATTTGGTAACTAGCTGCTTGGCTAGTTACCA
CCCACTAGCATAACCCCTTGGGGCCTCTAAACGGGTCTTGAGGGGTTTTTTGGTACCAAA
GGCTGAGCAAAGGG
